# Two Cerebrosides Isolated from the Seeds of *Sterculia lychnophora* and Their Neuroprotective Effect

**DOI:** 10.3390/molecules18011181

**Published:** 2013-01-17

**Authors:** Ru-Feng Wang, Xiu-Wen Wu, Di Geng

**Affiliations:** School of Chinese Materia Medica, Beijing University of Chinese Medicine, Beijing 100102, China

**Keywords:** *Sterculia lychnophora*, constituents, cerebrosides, neuroprotective effect

## Abstract

Two cerebrosides named 1-*O*-*β*-D-glucopyranosyl-(2*S*,3*R*,4*E*,8*Z*)-2-[(2-hydroxyoctadecanoyl)amido]-4,8-octadecadiene-1,3-diol (**1**) and soya-cerebroside I (**2**) were isolated from the seeds of *Sterculia lychnophora* for the first time. Their structures were completely characterized by spectroscopic methods including IR, MS and NMR. Compound **1** exhibited moderate neuroprotective effect against SH-SY5Y cell damage induced by hydrogen peroxide.

## 1. Introduction 

The dried and ripe seed of *Sterculia lychnophora* is a well-known Chinese medicine which has been used by Chinese people for a long history. It is mainly produced in Vietnam, Thailand, Malaysia, India, Indonesia as well as the southeastern part of China. This traditional drug is reputed for its prevention of, and as a remedy against pharyngitis. It has also been used for the treatment of tussis and constipation since ancient times in China [1]. According to the *Chinese Traditional Medicine Records*, the seed coat of *S. lychnophora* contains pentosan and viscous substances which belong to pectin acids and are mainly composed of galacturonic acid, arabinose, and galactose acetic acid. The perisperm of these seeds contains volatile oil, tragacanthgum and astringent substances. The kernel of the seeds contains fatty acids, spicy and bitter substances [2]. As was reported, galactose, rhamnose, sucrose, water-soluble polysaccharides such as PI, PII and PPIII, alkali-soluble polysaccharide PH, 2,4-dihydroxyl benzoic acid, *β*-sitosterol and daucosterol were also isolated from these seeds [3,4]. Pharmacological effects of the seeds of *S. lychnophora* were mainly reported in anti-inflammatory, analgesic, anti-bacterial and anti-viral scopes [5,6,7,8,9]. The present investigation provided two cerebrosides, which was the first report of cerebroside isolated from *S. lychnophora*, and one of them was demonstrated by MTT assay to possess moderate neuroprotective effect against SH-SY5Y cell damage induced by hydrogen peroxide.

## 2. Results and Discussion

### 2.1. Structure Identification of Compounds

**Figure 1:**
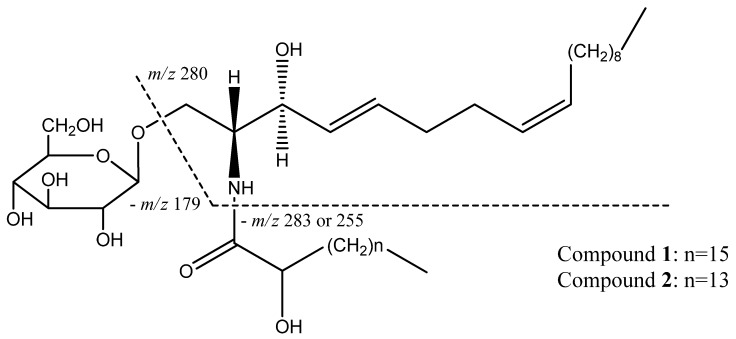
Structure and MS cleavage patterns of compounds **1** and **2**.

Compound **1** was obtained as a white powder. By thin layer chromatography, it was detected as a dark spot under 254 nm UV light and exhibited a brown spot after spraying with 10% H_2_SO_4_ in ethanol. Its IR spectrum revealed the characteristic absorption bands of hydroxyl (3415 cm^−1^), amide (1641 cm^−1^), aliphatic chain (2853 cm^−1^) and double bond (1537 cm^−1^) moieties. Both the ^1^H- and ^13^C-NMR data of this compound demonstrated the presence of an amide linkage, long aliphatic chains and a sugar residue. In the positive-SI-MS spectrum, *m/z* 742 indicated the pseudo-molecular ion [M+1]^+^, and *m/z* 562 indicated the fragment ion resulting from losing the sugar residue from the dehydrated pseudo-molecular ion. Another two ion peaks at *m/z* 280 and 262 were important for identification of the length of two aliphatic chains. Of these two signals, *m/z* 280 represented the ion generated by cleavage at the sites indicated in Figure 1, and *m/z* 262 indicated that the resulting *m/z* 280 ion was further dehydrated. All of the evidence above suggested the cerebroside nature of this compound. After thoroughly comparing the NMR and MS spectral data of compound **1** with those reported for the compound 1-*O*-*β*-D-glucopyranosyl-(2*S*,3*R*,4*E*,8*Z*)-2-[(2-hydroxyoctadecanoyl)amido]-4,8-octadecadiene-1,3-diol [9], the present compound was identified as the same one.

Compound **2** was determined as an analogue of compound **1**, which based on its spectral data only differed from compound **1** by two methylenes less in the aliphatic chain. After comparison of its spectral data with those reported in the literature [10], this compound was identified as soya-cerebroside I (Figure 1).

### 2.2. Neuroprotective Effect Against SH-SY5Y Cell Damage

**Figure 2:**
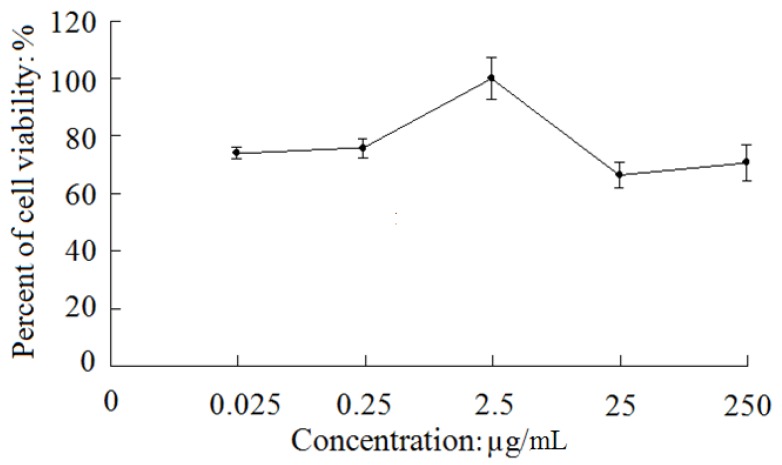
The viability of damaged SH-SY5Y cells induced by H_2_O_2_ after treatment with the test compounds.

Note: The bars indicate the SD of measurement (n = 3).

As shown in Figure 2, compound **1** exhibited a moderate protective effect against SH-SY5Y cell damage induced by hydrogen peroxide (H_2_O_2_). Up to 2.5 μg/mL, the percent of cell viability of the test groups including 0.025 μg/mL, 0.25 μg/mL, and 2.5 μg/mL dosage groups increased in a dose-dependent manner by 11.6% (*p* < 0.05), 13.6% (*p* < 0.01) and 37.6% (*p* < 0.01) compared to the model group (62.7% ± 5.1%), respectively, although they were not as high as that of the positive control (0.1 μg/mL EGF) group (141.2% ± 6.9%). However, the percent of cell viability decreased as the dosage further increased above 2.5 μg/mL, and were not significantly different from that of the model group (*p* > 0.05).

Cerebrosides are a kind of glycoside usually formed by sphingosine and a saccharide, reported to have neurotrophic and neuroprotective effects due to their property of easy penetration across the blood-brain barrier [11]. Compound 1 had a moderate neuroprotective effect on SH-SY5Y cells in a dose-dependent manner within the dosage range from 0.025 μg/mL to 2.5 μg/mL; however, as the dosage further increased above 2.5 μg/mL, the neuroprotective effect of this compound decreased instead. Moreover, compound 2 did not show a similar neuroprotective effect. The exact reason for this difference, given the structural similarity between the two compounds, is worthy of further study. 

## 3. Experimental

### 3.1. General Procedures

IR spectra were recorded on a Thermo Nicolet Nexus 470 FT-IR spectrophotometer. NMR spectra were determined on a JEOL JNM 300 (300 MHz for ^1^H-NMR and 75 MHz for ^13^C-NMR) spectrometer with the solvent as internal standard. SI-MS spectrum was measured on a Bruker APEX mass spectrometer and TOF-MS spectrum was obtained with a PE Q-STAR ESI-TOF-MS/MS spectrometer. The separation of two compounds was conducted on an Agilent 1100 HPLC apparatus equipped with G1315A DAD detector, G1312A Bin-Pump and HP Chemstation software. Absorbance of the samples in MTT assay was read on a 550 Automatic Microplate Reader. Silica gel (200–300 mesh) used in column chromatography was supplied by Qingdao Marine Chemistry Co. Ltd. (Qingdao, China) and analytical TLC was performed on self-made silica gel GF_254_ plates. Chromatographic separation reagent was of analytical grade and obtained from Beijing Chemical Factory (Beijing, China). Dulbecco’s Modified Eagle’s Medium (DMEM), fetal bovine serum (FBS) and trypsin were purchased from Gibco Laboratories (Life Technologies Inc., Grand Island, NY, USA). Epidermal Growth Factor (EGF), 3-(4,5-dimethylthiazol-2-yl)-2,5-diphenyltetrazolium bromide (MTT) and dimethyl sulfoxide (DMSO) were products of Sigma Chemical Co. (St. Louis, MO, USA). Cell culture flasks and 96-well plates were supplied by Corning Costar (Cambridge, MA, USA).

### 3.2. Plant Material

The experimental material was purchased from Anguo Chinese crude drug market in Hebei Province of China, and authenticated by Dr. Rufeng Wang as the seeds of *Sterculia lychnophora*. A voucher specimen has been deposited in the herbarium of School of Chinese Pharmacy, Beijing University of Chinese Medicine.

### 3.3. Extraction and Isolation

The seeds of *S. lychnophora* (2 kg) were powdered and extracted with 95% aqueous ethanol (8 L × 3 times, 1 h/time) under reflux. The extract solution was then concentrated *in vacuo* to yield an extract (240 g), which was suspended in water and partitioned successively with cyclohexane, ethyl acetate (EtOAc) and butanol (*n*-BuOH). Each solvent was evaporated *in vacuo* to afford cyclohexane extract (78 g), EtOAc extract (10 g) and *n*-BuOH extract (23 g). The EtOAc extract was subjected to column chromatography over a silica gel and eluted with CHCl_3_-MeOH to afford eight fractions. Fraction 6 gave compound **1** (8 mg) and **2** (3 mg) after separation by reverse phase semi-preparative liquid chromatography using a mixture of methanol and water as eluent.

### 3.4. Spectral Data of Compounds

Compound **1**: a white powder; IR (KBr) ν_max_: 3724, 3415, 2853, 1641, 1537, 1461, 1079, 961, 899, 718, 660 cm^−1^; Positive-SI-MS *m/z*: 742[M+1]^+^, 724[(M+1)−H_2_O]^+^, 562[[(M+1)−H_2_O]−162]^+^, 280[[(M+1)−283]−179]^+^, 262[[[(M+1)−283]−179]−H_2_O]^+^; ^1^H-NMR (CD_3_OD) *δ*: 5.74 (1H, *br*. d, *J* = 15.5 Hz, H-5), 5.49 (1H, dd, *J* = 15.5 Hz, 7.5 Hz, H-4), 5.44 (2H, t, *J* = 5.5 Hz, H-8, H-9), 4.26 (1H, d, *J* = 7.5 Hz, H-1″), 4.14 (1H, m, H-1a), 4.13 (1H, m, H-3), 3.99 (1H, m, H-2), 3.97 (1H, m, H-2′), 3.87 (1H, dd, *J* = 10.5 Hz, 1.5 Hz, H-6″a), 3.71 (1H, m, H-1b), 3.66 (1H, dd, *J* = 4.5 Hz, 1.5 Hz, H-6″b), 3.34 (1H, t, *J* = 6.5 Hz, H-3″), 3.27 (2H, m, H-4″, H-5″), 3.20 (1H, dd, *J* = 9.0 Hz, 7.5 Hz, H-2″), 2.06 (2H, m, H-6, H-7), 1.98 (1H, m, H-10), 1.70 (1H, m, H-3′a), 1.54 (1H, m, H-3′b), 1.40 (1H, m, H-4′), 1.33 (1H, m, H-11), 1.32 (12H, m, H-12~H-17), 1.30 (30H, m, H-5′~H-19′), 0.91 (6H, t, *J* = 7.0 Hz, CH_3_ × 2); ^13^C-NMR (CD_3_OD) *δ*: 177.2 (C-1′), 134.5 (C-5), 132.0 (C-8), 131.2 (C-9), 130.7 (C-4), 104.7 (C-1″), 78.0 (C-3″), 77.9 (C-5″), 75.0 (C-2″), 73.1 (C-2′), 72.8 (C-3), 71.6 (C-4″), 69.7 (C-1), 62.7 (C-6″), 54.6 (C-2), 35.9 (C-3′), 33.7 (C-6), 33.4 (C-7), 33.1 (C-10, C-4′), 30.9–23.8 (C-12~C-17, C-5′~C-19′), 33.0 (C-11), 14.5 (CH_3_). 

Compound **2**, a white powder; [*α*${]}_{D}^{20}$+12.5 (MeOH; *c* 0.50); TOF-MS (positive mode) *m/z*: 714 [M+1]^+^, 696 [(M+1)−H_2_O]^+^, 534 [[(M+1)−H_2_O]−162]^+^, 280 [[(M+1)−255]−179]^+^, 262 [[[(M+1)−255]−179] −H_2_O]^+^; ^1^H-NMR (CD_3_OD) *δ*: 5.75 (1H, *br*. d, *J* = 15.0 Hz, H-5), 5.50 (3H, m, H-4, H-8, H-9), 4.27 (1H, d, *J* = 7.8 Hz, H-1″), 4.15 (1H, m, H-1a), 4.00 (2H, m, H-3, H-2′), 3.87 (1H, dd, *J* = 11.1 Hz, 4.0 Hz, H-6″a), 3.72 (1H, m, H-1b), 1.70 (1H, m, H-3′a), 1.54 (1H, m, H-3′b), 1.40 (1H, m, H-4′), 1.30 (30H, m, H-5′~H-19′), 0.91 (6H, t, *J* = 7.0 Hz, CH_3_ × 2).

### 3.5. Preparation of Solutions

EGF was prepared at a concentration of 0.1 μg/mL, and was stored at −20 °C after membrane filter sterilization previous to use. Stock solution of compound **1** was prepared with culture medium (without FBS) at a concentration of 2.5 mg/mL. After membrane filter sterilization, this solution was stored at −20 °C until use. Final dilution was carried out with culture medium on the stock solution to afford a series of solutions of 250 μg/mL, 25 μg/mL, 2.5 μg/mL, 0.25 μg/mL and 0.025 μg/mL, respectively. MTT was dissolved in phosphate buffered solution (5 mg/mL), and the resultant solution was stored at −20 °C after membrane filter sterilization before use. MTT solution was diluted 10 times with PBS when added to 96-wells.

### 3.6. Cell Maintenance

Freeze-stored SH-SY5Y cells were recovered and cultured in DMEM containing 10% FBS, 100 U/mL penicillin, and 0.1 mg/mL streptomycin. Stock cultures were grown in 25 cm^2^ flasks in a humidified atmosphere of 5% CO_2_ in air at 37 °C. The culture medium was replaced every other day. When the cells reached 80% confluence, they were split 1:3 or 1:4 and passaged once every 3–4 days.

### 3.7. MTT Assay

Cells at logarithmic growth phase were washed twice with PBS and were removed from the flasks by incubating the monolayers with trypsin. The cells were collected into centrifuge tubes, spun at 700 rpm for 5 min, and the obtained pellet was resuspended in culture medium. Cells were seeded at a density of 5 × 10^4^ cells/mL onto 96-well plates, and in this way, each well was inoculated with 100 μL of cell suspension. The culture medium was removed after cells had been tightly attached to culture vessel growth surface, and cells were treated with 2 mM of H_2_O_2_ solution for 1 h. Then H_2_O_2_ solution was removed and each 200 μL of fresh culture medium (negative control group) and drug-containing medium (various dosage groups of compound **1** and the positive control group of EGF) was added to each hole. When cells were cultured in drug-containing medium for 24 h, the medium was completely transferred out and 100 μL of MTT solution (0.5 mg/mL) was added to each hole. MTT solution was removed carefully after four hours of incubation at 37 °C in culture hood, and each 150 μL of DMSO was added to each hole, then culture vessels were agitated on an orbital shaker for 10 min and absorbance was read on a microplate reader at 570 nm.

### 3.8. Data Analysis

The data obtained from triplicate wells were treated with statistical methods and expressed as means ± S.D. The statistical difference between two groups was investigated by an independent-samples t test. F test was also applied to the comparison of multiple mean. Statistical significance was set at the 0.05 level. 

## 4. Conclusions

This study first reported the presence of cerebrosides in the seeds of *S. lychnophora* and demonstrated the neuroprotective effect of one of these cerebrosides by bioactivity screen. Although the compound only exhibited moderate neuroprotective effect, this kind of compounds has already been highly expected in the treatment of mental dysfunctions such as Alzheimer’s disease. Therefore, our research at least provided a clue for discovery of new nootropics and we believe it will intrigue the interest of scientists to carry out in-depth investigation.
